# Hypoxia-induced overexpression of stanniocalcin-1 is associated with the metastasis of early stage clear cell renal cell carcinoma

**DOI:** 10.1186/s12967-015-0421-4

**Published:** 2015-02-12

**Authors:** Xin Ma, Liangyou Gu, Hongzhao Li, Yu Gao, Xintao Li, Donglai Shen, Huijie Gong, Shichao Li, Shaoxi Niu, Yu Zhang, Yang Fan, Qingbo Huang, Xiangjun Lyu, Xu Zhang

**Affiliations:** Department of Urology/State Key Laboratory of Kidney Diseases, Chinese PLA General Hospital/PLA Medical School, Beijing, China

**Keywords:** Clear cell renal cell carcinoma, Metastasis, Stanniocalcin-1, Hypoxia

## Abstract

**Background:**

Although metastasis of clear cell renal cell carcinoma (ccRCC) is predominantly observed in late stage tumors, early stage metastasis of ccRCC can also be found with indefinite molecular mechanism, leading to inappropriate clinical decisions and poor prognosis. Stanniocalcin-1 (STC1) is a glycoprotein hormone involved in calcium/phosphate homeostasis, which regulates various cellular processes in normal development and tumorigenesis. This study aimed to investigate the role and mechanism of regulation of STC1 in the metastasis of early stage ccRCC.

**Methods:**

STC1 mRNA and protein expression was determined in ccRCC surgical specimens, RCC cell lines, and human kidney tubule epithelial cell line HKC by real-time polymerase chain reaction (RT-PCR) and western blotting. Immunohistochemistry staining (IHC) and immunofluorescence were also used to examine the expression and localization of STC1 in ccRCC tissues and cancer cells. Knockdown and overexpression studies were conducted *in vitro* in RCC cell lines using small interfering RNAs (siRNA) and lentiviral-mediated gene delivery to evaluate the role of STC1 in cell proliferation, anchorage-dependent and independent growth, cell cycle control, and migration and invasion.

**Results:**

STC1 mRNA and protein expression were significantly up-regulated in tumors when compared with non-tumor tissues, with the greatest increase in expression observed in metastatic tissues. Clinicopathological analysis revealed that STC1 mRNA expression was associated with Fuhrman tumor grade (*P* = 0.008) and overall Tumor Node Metastasis (TNM) staging (*P* = 0.018). STC1 expression was elevated in T1 stage metastatic tumors when compared with localized tumors, and was positively correlated with average tumor diameter. Silencing of STC1 expression by Caki-1 and A498 resulted in the inhibition of cell proliferation, migration, and invasion, meanwhile down-regulation of STC1 impaired epithelial–mesenchymal transition (EMT) of ccRCC cell lines. Overexpression of STC1 in Caki-2 enhanced cell growth and proliferation but not migration and invasion. Further investigation identified hypoxia and HIF-1α as candidate regulators of STC1 expression.

**Conclusions:**

Our findings demonstrate a role for STC1 in metastasis of early stage ccRCC and suggest that STC1 may be a biomarker of potential value both for the prognosis of this disease and for guiding clinical decisions regarding surgical strategies and adjuvant treatment.

**Electronic supplementary material:**

The online version of this article (doi:10.1186/s12967-015-0421-4) contains supplementary material, which is available to authorized users.

## Background

Renal cell carcinoma (RCC), accounts for 2-3% of all adult malignancies, and is the second leading cause of death associated with urological malignant neoplasms. The most common histological subtype is clear cell RCC (ccRCC), accounting for approximately 80-90% of all RCCs [1]. The increasing number of patients presenting with lower stage disease in recent years is hypothesized to be the result of the more widespread use of multi-parametric imaging, which has resulted in an increase in the incidental discovery of renal tumors [2]. For early stage kidney tumors, partial nephrectomy is generally accepted as the standard approach for removing localized RCC and is associated with a good prognosis [3,4]. However, the prognosis of patients with metastatic renal cell carcinoma (mRCC) is extremely poor. A better understanding of the molecular mechanism underlying the pathogenesis of RCC is therefore needed to provide new biomarkers and therapeutic strategies for the treatment of this disease.

Stanniocalcin (STC) is a glycoprotein hormone involved in calcium and phosphate homeostasis, which was originally discovered as a secretory hormone of the corpuscles of Stannius, an endocrine gland of bony fish [5]. A human ortholog of fish STC, STC1, has been identified by molecular biology techniques. Mammalian STC1 is broadly expressed in various tissues including heart, lung, liver, adrenal gland, kidney, ovary, prostate, colon, bone and spleen [6-11]. STC1 appears to have multiple functions in physiological and pathological processes, including pregnancy [12], lactation [12], angiogenesis [13,14], organogenesis [15,16], cerebral ischemia [17], oxidative stress [18], and apoptosis [19,20]. Whereas the majority of studies have focused on the calcium-regulating functions of STC1, accumulating evidence suggests that STC1 may also play an important role in carcinogenesis. Increased STC1 expression has been observed in many types of cancer, including colorectal cancer, hepatocellular carcinoma [21,22], non-small cell lung cancer [23], ovarian cancer [10], breast carcinoma [24-27] and leukemia [28]. STC2 was identified from expressed sequence tag (EST) database searches for sequences related to STC1 [5]. STC2 is expressed in various tissues and associated with several types of cancer, and has been identified as a predictor for aggressiveness and overall patient survival in RCC [29].

Despite recent advances in our understanding of STC1, the expression pattern, clinical relevance, and biological role of this protein in ccRCC remains poorly understood. Recent studies indicate that STC1 expression is involved in the formation of tumor vasculature through up-regulation of vascular endothelial growth factor (VEGF) [13,14], STC1 can induce adaptive responses to hypoxia in human cancer cells through the regulation of hypoxia inducible factor-1-alpha (HIF-1α), a process that is closely associated with carcinogenesis and the progression of RCC [30,31].

In the present study, we examined STC1 mRNA and protein expression in ccRCC tissues and cell lines. We examined the correlations between STC1 expression and clinicopathological features, and evaluated the role of this protein in the metastasis of patients with early stage ccRCC. Moreover, we assessed the effects of knockdown and overexpression of STC1 *in vitro* on the proliferation, cell cycle progression, migration and invasion of RCC cells. Finally, we explored the possible mechanism of regulation of STC1 expression.

## Methods

### Ethics statement

Written informed consent was obtained from all patients prior to sample collection and the study was approved by the Protection of Human Subjects Committee of Chinese People’s Liberation Army General Hospital.

### Patients and tissue samples

Tissue specimens were obtained from patients with ccRCC who underwent partial or radical nephrectomy at the Chinese People’s Liberation Army (PLA) General Hospital (Beijing, China). A total of 122 patients with localized ccRCC and 24 patients with primary metastatic ccRCC were included in the study. We also included 48 adjacent non-tumorous kidney tissues from the localized group. All RCC cases were clinically and pathologically confirmed to be clear cell type and were staged according to the 2011 Union for International Cancer Control (UICC) TNM classification of malignant tumors. The nuclear grade was determined by the Fuhrman nuclear grading system. Macrovascular invasion displayed renal vein or inferior vena cava invasion which signified tumor malignancy. Specimens were immediately snap-frozen in liquid nitrogen after surgical removal. They were stored at −80°C until analysis. Clinicopathologic features for each of the subgroups are given in Additional file 1: Table S1.

### Cell lines, cell culture, and treatment with cobalt chloride

The ccRCC cell lines Caki-1, A498, Caki-2 as well as the human renal proximal tubular epithelial cell line HKC were preserved in our laboratory. According to the American Type Culture Collection, the Caki-1 cell line was metastatic cell, whereas the A498, Caki-2 cell lines were non-metastatic cells. The SN12-PM6 cell line was kindly provided by Dr. X.P. Zhang of the Department of Urology, Union Hospital (Wuhan, China). The cells were cultured in Dulbecco’s modified Eagle’s medium (HyClone), MEM-EBSS (HyClone), McCoy's 5A Medium (HyClone), DMEM/F12 (HyClone) with 10% fetal bovine serum (Gibco, USA), penicillin (100 U/ml), and streptomycin (100 U/ml). All cells were cultivated in a sterile incubator maintained at 37°C with 5% CO_2_. To induce chemical hypoxia, 250 or 500 μM of cobalt chloride (CoCl_2_) was added to the medium and the cells were treated for 24 hours.

### RNA isolation, reverse transcription and real-time PCR

The total RNA of cell lines and tissues were extracted using Trizol reagent (Invitrogen, Carlsbad, CA) and were reverse transcribed to cDNA via one-step RT-PCR kit (TransGen Biotech Co., Ltd, Beijing, China) according to the manufacturer’s instructions. Real-time quantitative polymerase chain reaction was performed in an Applied Biosystems 7500 Detection system with SYBR Green (TransGen Biotech Co., Ltd, Beijing, China). The relative mRNA levels of genes were normalized to peptidylprolyl isomerase A (PPIA) [32] using the 2^-ΔΔ^CT method. The primer sequences are given in Additional file 1: Table S2.

### Western blot analysis

Tissues and cells were lysed using RIPA lysis buffer (Beyotime) and the protein concentrations were quantified using BCA reagent (Applygen Technologies). Equivalent amounts of protein (50–80 μg) were separated by 10% SDS-polyacrylamide gels, and electro-transferred onto PVDF membranes. After blocking with 5% non-fat milk for one hour, the membranes were incubated with primary antibodies at 4°C overnight, followed by a 10 min wash with TBST, which was repeated three times. After this, the membranes were incubated with the corresponding secondary antibody for one hour at room temperature. In all specimens, rabbit anti-goat IgG-HRP, goat anti-mouse IgG-HRP and goat anti-rabbit IgG-HRP (ZSGB-BIO) were used as the secondary antibody at a dilution of 1:5000 respectively. Immunoreactive bands were visualized using an enhanced chemiluminescence (ECL) system (Thermo). The mean densities of the bands were represented as the OD in units per square millimeter and normalized to that of β-actin (Quantity One, version 4.6.2; Bio-Rad Laboratories, Inc., Hercules, CA, USA). We used specific primary antibodies detailed in Additional file 1: Table S3.

### Immunohistochemistry staining (IHC)

All of the samples were fixed in 10% neutral formalin. Sequential sections (4 μm thick) of ccRCC specimens and their corresponding adjacent normal renal tissue specimens were cut from wax blocks. A standard immunostaining procedure was then performed using a primary polyclonal goat antibody against human STC1 (1:100 dilution, Santa Cruz sc-14346). Afterwards, the slides were briefly counterstained with hematoxylin and aqueously mounted. Negative control was performed by replacing the primary antibody with goat serum. Immunostaining for STC1 protein was independently and blindly analyzed by two pathologists. According to standards from a previous study [10], tissues in which more than 10% of the cytoplasm was stained for STC1 protein were considered positive, and those with less than 10% staining were considered negative.

### RNAi knockdown

The small interference RNA molecules targeting human STC1 were designed and synthesized by GenePharma Co. (Shanghai, China). The knockdown efficiency of each siRNA was further confirmed by *in vitro* transfect to cells in our prior experiments. To enhance the silencing efficiency and reduce potential off-target effects, the three siRNAs (100 nM total, 33.3 nM each) were used simultaneously to knock down STC1 in cancer cell lines using lipofectamine 2000 (Invitrogen) according to the manufacturer’s recommendations. After transfection of siRNA or negative control for 48 h, the cells were harvested for further experiments. Sequences of siRNAs and negative control are provided in Additional file 1: Table S4.

### Construction of lentivirus (LV) for STC1 overexpression

For Plasmid construction, the coding domain sequence of STC1 was amplified from the pcDNA3.1-STC1 by high-fidelity PCR amplification. Primer sequences used were sense 5’-TGCTCTAGAATGCTCCAAAACTCAG-3’ and antisense 5’-CCGGAATTCTTATGCACTCTCATG-3’. The resulting fragment was inserted into the lentiviral vector PLV-EGFP (2A) Puro (Inovogen Tech. Co.) between XbaI and EcoRI to generate PLV-EGFP-STC1. The desired sequence was confirmed by direct DNA sequencing. PLV-EGFP empty vector or PLV-EGFP-STC1 was co-transfected with pH1 and pH2 into 293 T cells, respectively, by using lipofectamine 2000. After transfection for 48 hours, the viral supernatant was collected and filtered through a 0.45 μm filter and renamed as LV-EGFP and LV-STC1, respectively. The supernatants were used to infect Caki-2 cells with 6 μg/ml of polybrene (Sigma-Aldrich). After infection for 24 h, cells were cultured in selective medium (2 μg/ml puromycin) to select stable cell lines. STC1 expression was evaluated by RT-PCR and Western blotting. The Caki-2 cells infected with LV-EGFP and LV-STC1 were used for further analysis. Results of transfection efficiency are shown in Additional file 1: Figure S1.

### MTS assay

MTS is a tetrazolium compound, which can be bio-reduced by cells into a colored formazan product that is soluble in tissue culture medium. MTS assay was used to monitor cell proliferation by absorbance. Assays were performed as previously described [33]. The cells were seeded into 96-well plates (1000 cells/well) and cultured with 200 μl of 10% FBS/medium at 37°C in a 5% CO_2_ incubator. At the indicated time-points (24, 48, 72, 96 and 120 hours), 20 μl of CellTiter 96 Aqueous One Solution (Promega, Madison, WI) was added to each well and then incubated for 1 hour at 37°C. Absorbance was measured at 490 nm using an automatic enzyme-linked immunosorbent assay reader (BioTek Instruments).

### Anchorage-dependent and independent growth assays

For the anchorage-dependent growth assay, Caki-1 and A498 cells were separately seeded in six-well culture plates after transfection for 48 hours at a density of 1 × 10^3^ cells per well. Colony numbers were counted after they were fixed with methanol and stained with 0.2% crystal violet at 14 d. Caki-2 cells infected with LV-EGFP or LV-STC1 were also performed.

For the anchorage-independent growth assay, the cells that were suspended in 0.35% top agar were plated onto 0.7% base agar in six-well plates at a density of 1 × 10^3^ cells. After incubating for three to four weeks, colonies were scored.

### Cell cycle analysis

After transfection for 48 hours, the Caki-1 and A498 cells were collected, washed with phosphate-buffered saline, and then fixed in 70% ice-cold ethanol overnight. Then the cells were stained with propidium iodide (Beyotime, Shanghai, China) according to the manufacturer’s instructions. DNA analysis was performed using a FACS-Calibur (BD Biosciences). Each experiment was performed in triplicate and repeated three times. Caki-2 cells infected with LV-EGFP or LV-STC1 were prepared for cell cycle analysis using the same method.

### Cell migration and invasion assay

Cell migration and invasion assays were performed in 24-well plates using Boyden chambers containing Transwell (Corning, NY) membrane filter inserts with a pore size of 8 μm. For invasion assay, the membrane undersurface was coated with 30 μl ECM gel from Engelbreth-Holm-Swarm mouse sarcoma (BD Biosciences) mixed with serum-free medium in 1:5 dilution for 4 h at 37°C. After transfection for 48 h or infection with LV-EGFP or LV-STC1, 1 × 10^5^ cells in serum-free medium were added to the upper chambers, and the bottom chambers were filled with 500 μl medium containing 10% FBS. After 12 (migration) or 24 (invasion) hours at 37°C, non-migrating cells on the upper surface of the filters were gently scraped. Invading and migrating cells were fixed and stained with 0.1% crystal violet. The cells were randomly counted under a microscope in the five representative areas on each plate. All assays were performed independently three times.

### Wound healing assay

After transfection for 48 h or infection with LV-EGFP or LV-STC1, cell lines Caki-1, A498 and Caki-2 in interfered group and negative control group were seeded on 6-well plates with fresh medium containing 10% FBS. Confluent monolayer cells were scratched using a sterile 200 μL pipette. Photos of the wound were taken at different time points (0, 12, and 24 h after scratching). The coverage of the scraping area was measured at three positions for each replicate. Experiments were performed in triplicate.

### Cell immunofluorescence and imaging

Immunofluorescence staining was performed as previously described [34]. The cells were seeded and grown on glass coverslips 24 h prior to experiment. After fixation with 4% paraformaldehyde-PBS for 15 min, cells were then permeabilized in 0.5% Triton X-100 in PBS and sequentially blocked with 3% bovine serum albumin for 30 min. The coverslips were incubated at 37°C for 1 h with primary antibodies recognizing STC1 (Santa Cruz), E-Cadherin (Cell Signaling), N-Cadherin (Abcam), Vimentin (Cell Signaling), α-smooth muscle actin (α-SMA) (Abcam) separately, then incubated with fluorescein isothiocyanate (FITC)-conjugated goat anti-rabbit or goat anti-mouse IgG at 1:200 dilution. Nuclei were stained with 0.2 mg/mL DAPI for 15 min at 37°C. Immunofluorescence was visualized under an Olympus fluorescence microscope and image-captured. Figures were assembled using OLYMPUS Fluoview FV1000 (version 1.6). The fluorescent microscope images were assessed using Image-Pro Plus 6.0 software, and the integral optical density (IOD) of each photograph was collected. The primary antibodies used in this study are given in Additional file 1: Table S3.

### Statistical analysis

All data were analyzed using the SPSS statistical software 13.0(SPSS Inc., Chicago, IL), and *P* < 0.05 was considered statistically significant. All continuous data were tested for normality with the Kolmogorov-Smirnov test. Student *t* test or one-way ANOVA was used to compare normally distributed variables. The Mann–Whitney U or Kruskal-Wallis test was used to compare continuous variables not conforming to the assumptions of normality. The correlation of two variables was analyzed by linear regression analysis.

## Results

### STC1 mRNA and protein expression in ccRCC tissues and RCC cell lines

**Figure 1:**
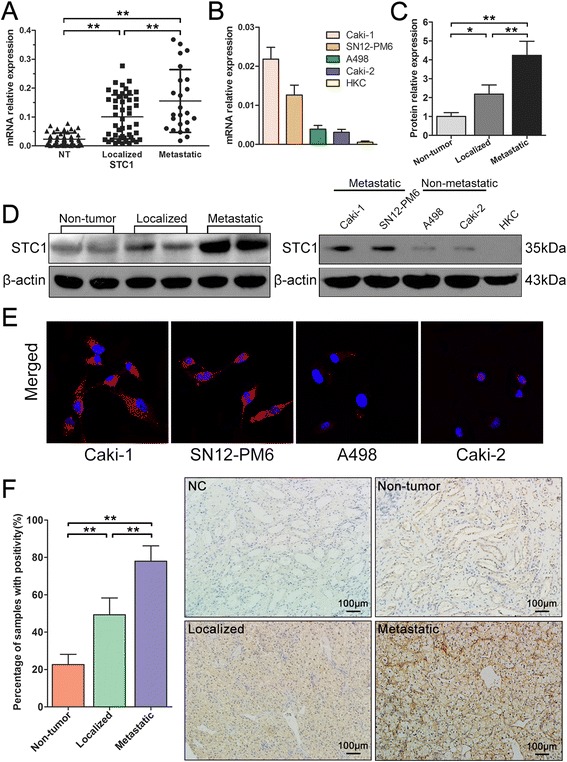
**Expression of stanniocalcin-1 (STC1) in clinical samples and renal cell carcinoma (RCC) cell lines. (A)** The mRNA level of STC1 was significantly up-regulated in clear cell renal cell carcinoma (ccRCC) tissues compared with adjacent normal renal tissues, with the greatest expression observed in metastatic tissues. **(B)** The mRNA expression of STC1 was up-regulated in RCC cell lines compared with HKC-renal tubular epithelial cell line, with the greatest expression observed in metastatic cells. **(C) (D)** Western blotting showed the changes of protein expression were consistent with these of mRNA in clinical samples and RCC cell lines. **(E)** Immunofluorescence analysis revealed a significant increase in the intensity of STC1 staining in the cytoplasm of metastatic cell lines. **(F)** Representative images of STC1 immunohistochemistry staining in non-tumor (NT) tissues, localized and metastatic ccRCC tissues. Scale bar, 100 μm. Quantitative analysis of STC1 protein levels in the cytoplasm in 10 sets of paired NT tissues, localized and metastatic ccRCC tissues (n = 10). *,*P* < 0.05; **,*P* < 0.01.

The expression of STC1 in ccRCC tissues and RCC cell lines was evaluated by RT-PCR and western blotting. Twenty four primary metastatic and 48 primary non-metastatic ccRCC tissue samples from patients were matched 1:2 according to patient age and gender. Results indicated that the mRNA level of STC1 was significantly up-regulated in ccRCC tissues (both in localized, *P* < 0.01, and metastatic tissues, *P* < 0.01; Figure 1A) compared with adjacent normal renal tissues, with the greatest expression observed in metastatic tissues (*P* < 0.01, Figure 1A). Elevated expression of STC1 protein was also observed in tumor tissues when compared with non-tumor tissues, again with the highest expression observed in metastatic tumors (Figure 1C and D). Immunohistochemical staining demonstrated that the STC1 protein was predominantly localized in the cytoplasm of both renal cancer tissues and normal renal tissues. Cytoplasmic levels of this protein were significantly elevated in 10 metastatic ccRCC tissue samples, when compared with 10 localized ccRCC tissues and 10 normal renal tissues, a finding that was in agreement with our results from RT-PCR and western blotting analysis (Figure 1F).

To evaluate STC1 expression in ccRCC cell *in vitro*, metastatic RCC cell lines (Caki-1, SN12-PM6), non-metastatic cell lines (A498, Caki-2) and human renal proximal tubular epithelial cell line HKC were analyzed by RT-PCR and western blotting. Levels of STC1 mRNA and protein were significantly up-regulated in ccRCC cells when compared with normal epithelial cells (Figure 1B and D). Furthermore, expression of STC1 mRNA and protein was found to be higher in metastatic RCC cells when compared with non-metastatic cells. Immunofluorescence analysis also revealed a significant increase in the intensity of STC staining in the cytoplasm of metastatic cell lines (Figure 1E, Additional file 1: Figure S1E).

As shown in Table 1, STC1 mRNA levels were significantly associated with Fuhrman tumor grade (*P* = 0.008) and overall TNM staging (*P* = 0.018).

**Table 1:** **STC1 mRNA expression in relation to ccRCC clinicopathologic features**

**Clinicopathologic features**	**No. n = 146**	**STC1 mRNA expression mean ± SD**	***P*** **value**
Age, y			0.960
<60	102	0.1263 ± 0.0094	
≥60	44	0.1254 ± 0.0130	
Gender			0.147
Male	100	0.1336 ± 0.0096	
Female	46	0.1094 ± 0.0128	
Tumor size, cm			0.670
≤7	121	0.1245 ± 0.0083	
>7	25	0.1333 ± 0.0209	
Fuhrman tumor grade			0.008*
I-II	116	0.1156 ± 0.0082	
III-IV	30	0.1660 ± 0.0190	
T staging			0.173
T1 + T2	123	0.1195 ± 0.0084	
T3	23	0.1478 ± 0.0178	
Overall TNM staging			0.018*
I-II	105	0.1145 ± 0.0089	
III-IV	41	0.1553 ± 0.0150	
Necrosis			0.510
Yes	74	0.1311 ± 0.0117	
No	72	0.1208 ± 0.0102	
Microvascular invasion			0.188
Yes	22	0.1503 ± 0.0190	
No	124	0.1217 ± 0.0085	

*Statistically significant (*P* < 0.05).

### Elevated expression of STC1 is associated with metastasis of ccRCC at T1 stage

**Figure 2:**
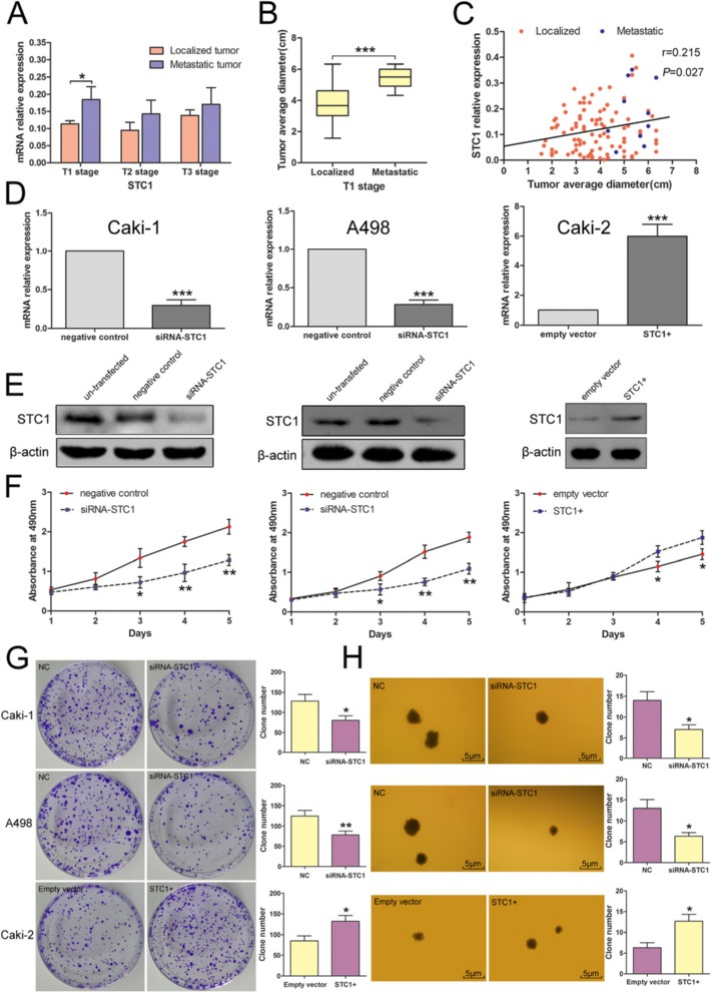
**Elevated expression of STC1 is associated with metastasis of T1 stage ccRCC. Effects of STC1 interferences on cell proliferation. (A)** Analysis showing higher STC1 expression in T1 stage metastatic tumors when compared with localized tumors. **(B)** Analysis showing significantly larger average diameter in metastatic tumors (5.433 ± 0.644 cm, n = 10) when compared with localized tumors (3.833 ± 1.187 cm, n = 96). **(C)** Positive correlation of STC1 mRNA levels and average tumor diameter in T1 stage (n = 106, r = 0.215, p = 0.027). **(D) (E)** Alteration for STC1 mRNA and protein levels in Caki-1-Metastatic cell line, A498-Non-metastatic cell line and Caki-2-Non-metastatic cell line cells after knockdown or overexpression. **(F)** MTS assay showed STC1 knockdown reduced the proliferation of Caki-1 and A498 cells, while STC1 overexpression promoted the proliferation of Caki-2 cells. **(G) (H)** Effects of STC1 interferences on anchorage-dependent and independent growth of RCC cells. *,*P* < 0.05; **,*P* < 0.01; ***,*P* < 0.001.

Given that small renal mass sometimes accompanies metastasis [35], we examined whether STC1 is associated with the metastasis of early stage ccRCC. A comparison of STC1 mRNA expression between localized and metastatic tumors, revealed that only T1 stage tumors exhibited a statistically significant difference in STC1 expression when compared with other tumor stages (*P* = 0.021, Figure 2A).

Given that tumor size has been reported to be significantly associated with risk of metastasis [36], we next analyzed the average tumor diameter of localized and metastatic T1 stage tumors. As shown in Figure 2B, the average diameter of metastatic tumors (5.433 ± 0.644 cm, n = 10) was larger than that of localized tumors (3.833 ± 1.187 cm, n = 96, *P* < 0.001). Furthermore, analysis of the relationship between STC1 expression and T1 stage ccRCC tumor size (Figure 2C) revealed that STC1 mRNA levels correlated with average tumor diameter (n = 106, r = 0.215, *P* = 0.027).

### STC1 promotes cellular proliferation

Because tumor size is primarily determined by the proliferation of cancer cells, we sought to verify whether the larger tumor size which was observed in metastatic ccRCC was a result of elevated STC1 expression. The knockdown of STC1 by transfection of specific siRNA was performed in both Caki-1 and A498 cells. RT-PCR and western blotting analysis confirmed that STC1 mRNA and protein levels in the siRNA group were decreased after transfection for 48 h (Figure 2D and E). Confirmation of STC1 overexpression in Caki-2 cells following lentiviral infection with LV-STC1 was also verified by RT-PCR and western blotting (Figure 2D and E). MTS and anchorage-dependent growth assays showed that knockdown of STC1 significantly reduced the proliferation of Caki-1 and A498 cells (*P* < 0.05, Figure 2F and G), while STC1 overexpression promoted the proliferation of Caki-2 cells (*P* < 0.05, Figure 2F and G). Moreover, silencing of STC1 in Caki-1 and A498 cells inhibited anchorage-independent growth, while STC1 overexpression in Caki-2 cells promoted this behavior (*P* < 0.05, Figure 2H).

### STC1 accelerates G1/S transition in cell cycle

**Figure 3:**
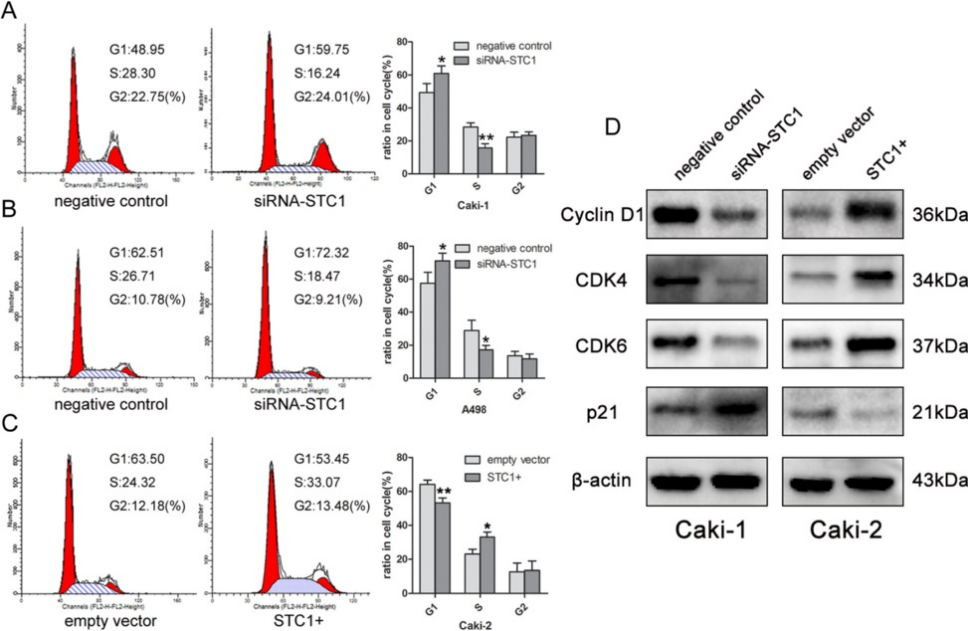
**Effect of STC1 interferences on cell cycle. (A) (B)** Knockdown of STC1 led to G1 restoration compared with negative control group in Caki-1 and A498 cells. **(C)** Overexpression of STC1 induced G1-S phase transition compared to empty vector group in Caki-2 cells. **(D)** Western blotting results for the expression alteration of several key cell cycle regulatory factors. *,*P* < 0.05; **,*P* < 0.01.

To evaluate whether STC1 modulates proliferation through an effect on the cell cycle, we conducted cytometry assays. Knockdown of STC1 in Caki-1 and A498 cells resulted in a higher percentage of cells in G1 phase and a concomitantly lower percentage of cells in S phase, when compared with controls (Figure 3A and B). In contrast, STC1 overexpression in Caki-2 cells resulted in the rapid passage of cells through the G1/S checkpoint (*P* < 0.05, Figure 3C). Therefore, STC1 promotes cell cycle progression and this may in part explain the proliferative effect of STC1 on ccRCC cells. To investigate the mechanism underlying this change in cell cycle progression, we examined expression of several key cell cycle regulatory factors by western blotting. As shown in Figure 3D, STC1 knockdown in Caki-1 cells significantly reduced the expression of cyclin D1, cyclin-dependent kinase 4 (Cdk4) and cyclin-dependent kinase 6 (Cdk6), and enhanced the expression of cyclin-dependent kinase inhibitor p21. Conversely, overexpression of STC1 in Caki-2 cells elevated the expression of cyclin D1, Cdk4 and Cdk6, and suppressed expression of p21.

### STC1 regulates the migration and invasion of ccRCC cells *in vitro*

**Figure 4:**
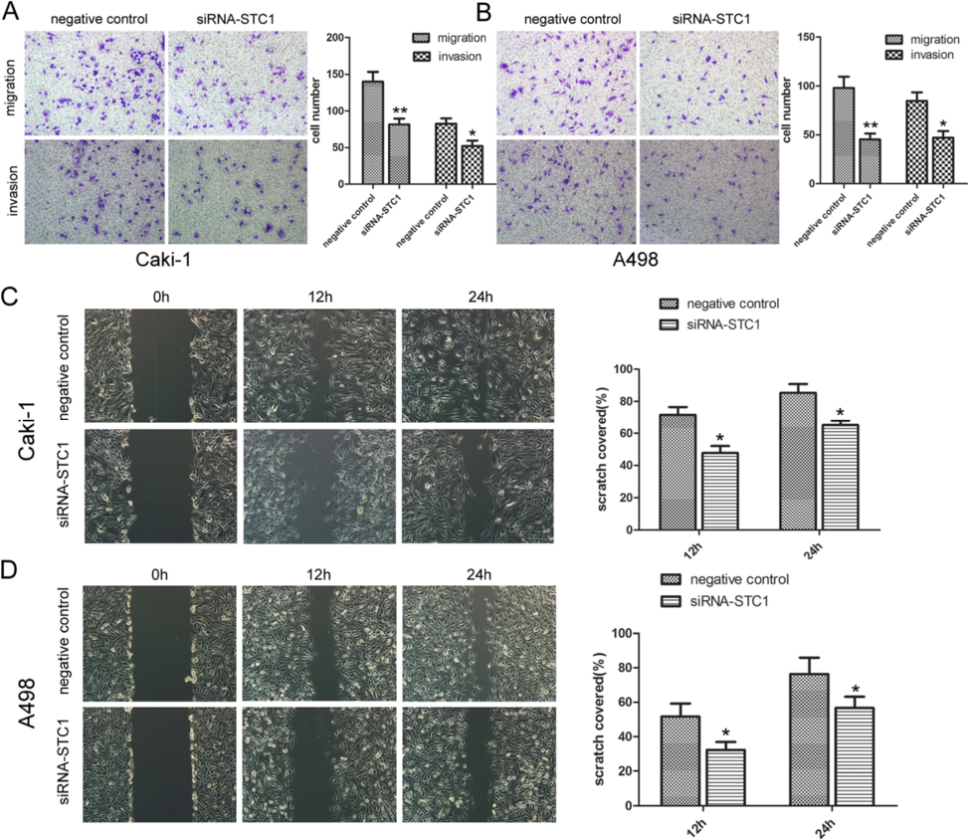
**Ability of STC1 in influencing the migration and invasion of ccRCC cells** ***in vitro*** **. (A) (B)** Representative photographs of Transwell assays (magnification, ×100) in Caki-1 and A498 cells. **(C) (D)** Confluent monolayer cells were scratched using a sterile 200 μL pipette. Photos of the wound were taken at different time points (0, 12, and 24 h after scratching). STC1 knockdown impaired the cell mobility in Caki-1 and A498 cells. Each experiment was performed triplicates. *,*P* < 0.05; **,*P* < 0.01.

Next, we sought to determine the role of STC1 in the invasion and migration of ccRCC cells. Transwell assays were performed to evaluate the ability of A498 and Caki-1 cells to permeate the membrane (invasion assay with Matrigel) following knockdown of STC1. Experiments revealed that the migration and invasion of cancer cells were significantly decreased following knockdown of STC1 when compared with controls (*P* < 0.05, Figure 4A and B). However, STC1 overexpression had no effect on the migration and invasive of Caki-2 when compared with empty vector group (Additional file 1: Figure S1A).

Wound healing assay revealed that knockdown of STC1 significantly reduced the cell migration at different time points in Caki-1 (*P* < 0.01, Figure 4C) and A498 cells (*P* < 0.01, Figure 4D). As with transwell assays, however, overexpression of STC1 had no effect on the migratory ability of Caki-2 cells (Additional file 1: Figure S1B).

**Figure 5:**
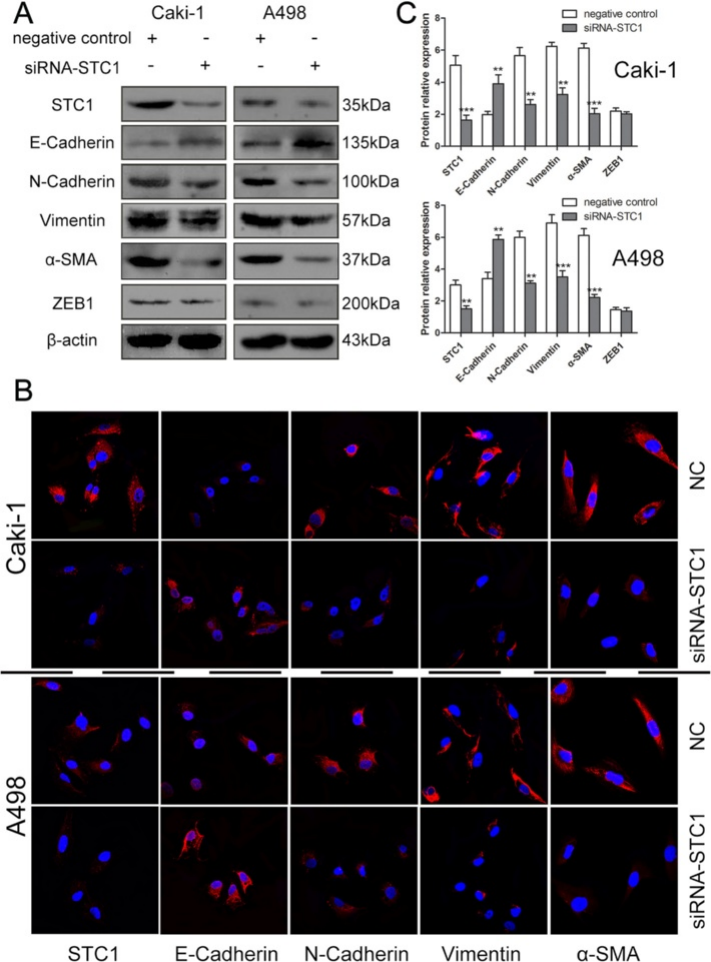
**Knockdown of STC1 impairs epithelial-mesenchymal transition (EMT) of Caki-1 and A498 cells. (A) (B)** Forty-eight hours after transfection, a series of EMT-related markers, including the mesenchymal markers N-cadherin, Vimentin, α-SMA, ZEB1, and the epithelial marker E-cadherin were determined by Western blotting and immunofluorescence staining (magnification, ×600) Red, CF555; blue, DAPI. **(C)** Western blotting results for the protein expression alteration of several EMT-related markers. **,*P* < 0.01; ***,*P* < 0.001.

Since epithelial–mesenchymal transition (EMT) influences ccRCC progression, we examined the expression of a series of EMT-related markers in Caki-1 and A498 cells. Expression of the mesenchymal markers N-cadherin, Vimentin, α-SMA, ZEB1, the epithelial marker E-cadherin, and the matrix metalloproteinases MMP2 and MMP9 were determined by western blotting, RT-PCR and immunofluorescence. Expression analysis revealed that N-cadherin, Vimentin and α-SMA levels were reduced and that E-cadherin was up-regulated in Caki-1 and A498 cells after knockdown of STC1 (*P* < 0.01, Figure 5, Additional file 1: Figure S1E). Also, MMP9 was found to be markedly down-regulated in both Caki-1 and A498 cells (*P* < 0.05, Additional file 1: Figure S1D). Considering the important roles of these markers, the effect of STC1 on these EMT markers has suggested its possible role in tumor progression.

### Hypoxia induces STC1 expression

**Figure 6:**
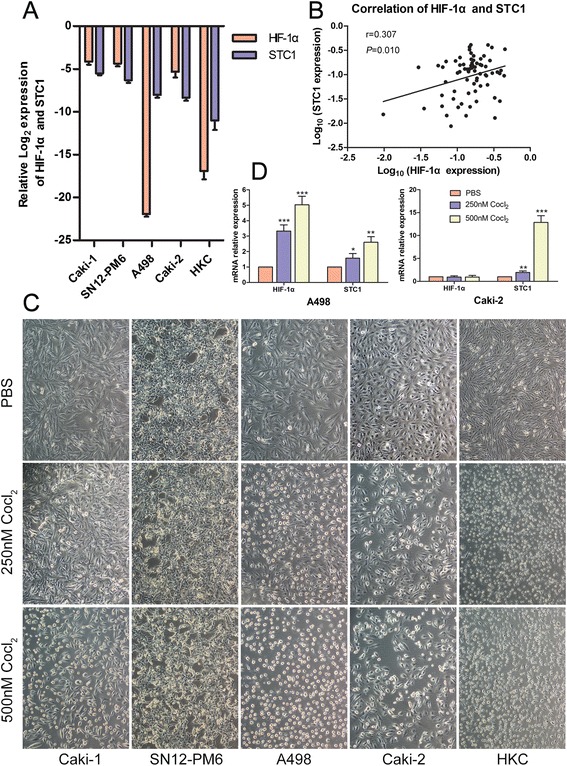
**Hypoxia induces STC1 expression. (A)** The mRNA expression trend of STC1 and hypoxia inducible factor-1-alpha (HIF-1α) in ccRCC cell lines and HKC cells. **(B)** The association between STC1 and HIF-1α mRNA expression in clinical samples. **(C)** Representative images showed the morphological changes of RCC cell lines and HKC cells after CoCl_2_ treatment (magnification, ×100). **(D)** The mRNA level changes of STC1 and HIF-1α in A498 and Caki-2 cells after CoCl_2_ treatment. *,*P* < 0.05; **,*P* < 0.01; ***,*P* < 0.001.

The level of STC1 mRNA correlated closely with that of HIF-1α in ccRCC cell lines, with the exception of A498 (Figure 6A). Furthermore, a positive correlation was observed between the expression of STC1 and HIF-1α mRNA in clinical samples (n = 70, r = 0.307, *P* = 0.010, Figure 6B). This result suggested that HIF-1α may regulate STC1 expression.

To confirm this hypothesis, four RCC cell lines (Caki-1, SN12-PM6, A498 and Caki-2) and human kidney tubule epithelial cell line HKC were treated with cobalt chloride (CoCl_2_), which mimics the hypoxic state. After CoCl_2_ treatment, morphological changes were observed in all cells analyzed, with the greatest changes observed for HKC cells that were more sensitive to hypoxia than RCC cells. Cell shrinkage and necrosis were observed and cell density decreased. The morphological damage aggravated with the increase of the dose of CoCl_2_ (Figure 6C). Under hypoxic conditions, expression of both STC1 and HIF-1α mRNA were up-regulated in A498 cells, while only STC1 was up-regulated in Caki-2 cells (Figure 6D).

## Discussion

Partial nephrectomy is recommended as a standard treatment for localized ccRCC in early stage [4], and resection of primary renal cell carcinoma can be curative. However, distant metastasis in patients with ccRCC remains the main cause of treatment failure and death from cancer [37]. This highlights the need to identify those patients most likely to develop metastasis who may benefit from radical nephrectomy and adjuvant treatment. Currently, post-operative histopathological parameters are commonly used to stratify patients [38]. But these variables are not considered entirely reliable, and further improvement in preoperative prediction is warranted. Thus, a molecular biomarker, which can accurately forecast the risk of metastasis in patients with early stage RCC, is required to help guide clinical decisions.

Mammalian STC1 is a ubiquitously expressed and secreted phosphoglycoprotein functioning in an autocrine/paracrine manner [20]. Studies have demonstrated that STC1 is involved in calcium and phosphate homeostasis and that it may have cytoprotective and anti-inflammatory functions by inhibiting reactive oxygen species [5,20,39-42]. Recently, a series of studies revealed the involvement of STC1 in cancer progression and metastasis [10,22,26,27,43,44], which motivated us to focus on the role of STC1 in ccRCC development and progression. Until now, the exact role of STC1 in ccRCC has never been characterized. Yang et al. [45] observed that HIF-1α may participate in the malignant proliferation of renal carcinoma cells by promoting STC1 accumulation or by down-regulating Ca^2+^, but such an effect may gradually attenuate because of the inhibitory effect of STC1 on HIF-1α.

In this work, we found that both STC1 mRNA and protein levels were up-regulated in ccRCC tissues when compared to their matched adjacent non-tumor counterparts. More importantly, STC1 expression levels were elevated to a greater extent in ccRCC tissues associated with distant metastasis. For further confirmation, we examined STC1 expression in ccRCC cell lines. In agreement with our tissue findings, STC1 was found to be highly expressed in ccRCC cell lines, with the greatest levels of STC1 expression being observed in metastatic cell lines. These data are consistent with observations reported for the majority of malignancies [10,22,23,46,47], although with the exception of cervical cancer [48].

Our analysis of STC1 mRNA expression in ccRCC tumors of different stages revealed that STC1 expression was up-regulated in T1 stage metastatic tumors when compared with localized tumors. Also, STC1 mRNA expression in T2 and T3 metastatic tumors was much higher than that in localized tumors, which barely achieved statistical significance. Previous studies have reported that tumor size is a risk factor in the metastasis of T1 stage RCC, with the synchronous metastasis rate increasing with tumor size [49,50]. In our study, we identified a positive correlation between STC1 expression and average tumor diameter.

Because tumor size primarily determined by the proliferative ability of tumor cells, we examined whether the larger tumor size observed in metastatic ccRCC was a result of high levels of STC1 expression. In agree with previous studies [10,14,44], knockdown of STC1 decreased proliferation and led to G1 arrest in ccRCC cell lines (Caki-1 and A498). Conversely, overexpression of STC1 in Caki-2 enhanced proliferation and induced G1/S transition. Furthermore, we found that knockdown or overexpression of STC1 caused a marked alteration in the expression of cyclin D1, Cdk4, Cdk6 and p21, all of which are key proteins in the regulation of cell cycle control. Liu et al. [10] reported that STC1 overexpression increased the expression of cyclin A, cyclin B1, CDK2, and a short cyclin E isoform in ovarian cancer cells, and it is possible that the elevated expression of these proteins is required for the regulation of G1 to S phase transition. However, Murai et al. [27] reported that overexpression of STC1 had no effect on the proliferation of MDA-MB-231 human breast cancer cells. Inconsistencies regarding the effect of STC1 on cancer cell proliferation may be owing to the different mechanisms exploited by tumor cells to promote growth and survival. Taken together, our data indicate that STC1 drives tumor cell growth and proliferation, and is consistent with the tumor growth, and ultimately the metastasis observed for early stage ccRCC.

Invasion and migration are two key features of metastatic malignancies, and are thought to increase the metastatic potential of cancer cells. In the current study, we found that STC1 silencing suppressed the migration and invasion of renal cancer cells, whereas overexpression of STC1 had no effect on these aspects of cell behavior. A possible reason for the lack of a migratory phenotype associated with STC1 overexpression is that basal STC1 expression in Caki-2 cells already exceeds the threshold required for these functions. EMT has been defined as a possible mechanism of metastasis; it can transform epithelial tumor cells and confer the mesenchymal characteristics that would facilitate the dissemination of these cells, leading to metastases. Because it has previously been reported that EMT influences ccRCC progression [51], we examined the expression of a series of EMT-related markers. Expression of the mesenchymal markers N-cadherin, Vimentin, α-SMA and MMP9, and the epithelial marker E-cadherin were altered as a result of STC1 silencing in Caki-1 and A498 cells. Law et al. [14] showed that overexpression of STC1 in HUVEC cells stimulated migration, and induced Vimentin expression. The effects of STC1 induced HUVEC remodeling were further exemplified by a concomitant increased in the expression and activity of MMP2 and a decrease in its endogenous inhibitor, tissue inhibitor of matrix metalloproteinase (TIMP1). Murai et al. [27] have reported that secreted STC1 promotes the metastatic potential of breast cancer cells via activation the of PI3K/AKT signaling axis. Other research has identified STC1 as a mediator of metastasis associated with PDGF receptor function in the colorectal cancer setting [44]. PDGF-stimulated fibroblasts were shown to increase the migration and invasion of co-cultured colorectal cancer cells in an STC1-dependent manner. In an orthotopic mouse model of colorectal cancer, cells from tumors formed in the presence of STC1-deficient fibroblasts displayed reduced intravasation, resulting in fewer and smaller distant metastases [44]. These data indicate that the increase in metastatic potential and malignancy promoted by STC1 in ccRCC cells is linked to EMT-related markers such as E-cadherin, N-cadherin, Vimentin, α-SMA and MMP9 which function to degrade the vascular wall. The result was consistent with the finding that higher STC1 expression in ccRCC was associated with higher Fuhrman grade and overall TNM staging. In mammals, an important role of STC1 has also been suggested during gestation, in the placenta [52], ovaries [12] and and endometrium [53,54]. Like cancer cells, placental cells are characterized by invasive nature and migratory properties which may partly support the function of STC1 in ccRCC cells.

A number of studies have reported that STC1 is associated with hypoxia and the major hypoxia-regulated factor HIF-1α [20,30,31]. Law et al. [31] have shown that hypoxia-inducible factor-1 (HIF-1) binds to human STC1 gene promoter and transactivates STC1 expression. In this study, hypoxic conditions were found to up-regulate mRNA for both STC1 and HIF-1α in von Hippel-Lindau (VHL) -deficient A498 cells, whereas only STC1 mRNA was up-regulated in Caki-2 cells. The reason for this may be the differential expression of VHL protein in these cells, given that wild-type VHL protein acts to prevent HIF-1α accumulation. Perhaps, these are some other factors regulating STC1 expression in hypoxia condition may account for the inconsistency of STC1 and HIF-1α in Caki-2 cells. HIF-1 plays a central role in RCC carcinogenesis and progression [55,56]. In the current study, we show for the first time the relationship between STC1 and hypoxia both in clinical samples of ccRCC and *in vitro* in ccRCC cell lines. Our data provide an insight into the possible role of STC1 in renal cancer cell survival under hypoxic conditions.

## Conclusions

To the best of our knowledge, this is the first time that STC1 has been implicated as a biomarker associated with the progression and metastasis of ccRCC. Here, we demonstrate that STC1 is associated with the metastasis of early stage ccRCC by regulating the proliferation, cell cycle, migration and invasion of tumor cells. This new biomarker may be helpful both for guiding clinical decisions when considering surgical options and adjuvant treatment, and for the prediction of ccRCC outcome. Hypoxia and HIF-1α were identified as candidate regulators of STC1 expression. Future experiments and research should focus on the biological roles of STC1 *in vivo* and the underlying mechanism of its action.

## Additional file

Additional file 1: Figure S1. STC1 overexpression had no effect on cell migration and invasion. Results of transfection efficiency. (A) Representative view of Transwell assays (magnification, ×100) for STC1 overexpression and empty vector control groups in Caki-2 cells. (B) For wound healing assay, STC1 overexpression had no effect on cell mobility in Caki-2 cells. (C) Representative fluorescence (GFP) micrographs of Caki-2 cells infected with LV-EGFP and LV-STC1 (magnification, ×100). (D) The matrix metalloproteinases MMP2 and MMP9 were examined by real-time polymerase chain reaction. (E) Results of relative fluorescence intensity. **Table S1.** Clinicopathologic features for each of the subgroups. **Table S2.** Real-time PCR primers. **Table S3.** Antibodies features. **Table S4.** Sequences of siRNAs.

## Abbreviations

ccRCC: Clear cell renal cell carcinoma
STC1: Stanniocalcin-1
RT-PCR: Real-time polymerase chain reaction
siRNA: Small interfering RNA
EMT: Epithelial-mesenchymal transition
mRCC: Metastatic renal cell carcinoma
VEGF: Vascular endothelial growth factor
HIF-1α: Hypoxia inducible factor-1-alpha
α-SMA: α-smooth muscle actin
MMP: Matrix metalloproteinase
VHL: Von Hippel-Lindau
