# Supplementary material for: Hypoxia-induced overexpression of stanniocalcin-1 is associated with the metastasis of early stage clear cell renal cell carcinoma
Source: J Transl Med. 2015 Feb 12;13:56. doi: 10.1186/s12967-015-0421-4 (PMC4337255; doi:10.1186/s12967-015-0421-4)
Supplement: Additional file 1: Figure S1. — STC1 overexpression had no effect on cell migration and invasion. Results of transfection efficiency. (A) Representative view of Transwell assays (magnification, ×100) for STC1 overexpression and empty vector control groups in Caki-2 cells. (B) For wound healing assay, STC1 overexpression had no effect on cell mobility in Caki-2 cells. (C) Representative fluorescence (GFP) micrographs of Caki-2 cells infected with LV-EGFP and LV-STC1 (magnification, ×100). (D) The matrix metalloproteinases MMP2 and MMP9 were examined by real-time polymerase chain reaction. (E) Results of relative fluorescence intensity. Table S1. Clinicopathologic features for each of the subgroups. Table S2. Real-time PCR primers. Table S3. Antibodies features. Table S4. Sequences of siRNAs. [file 12967_2015_421_MOESM1_ESM.pdf]

Additional files

Additional file 1: Figure S1. STC1 overexpression had no effect on cell migration and invasion. Results of transfection efficiency. (A) Representative view of Transwell assays (magnification, ×100) for STC1 overexpression and empty vector control groups in Caki-2 cells. (B) For wound healing assay, STC1 overexpression had no effect on cell mobility in Caki-2 cells. (C) Representative fluorescence (GFP) micrographs of Caki-2 cells infected with LV-EGFP and LV-STC1 (magnification, ×100). (D) The matrix metalloproteinases MMP2 and MMP9 were examined by real-time polymerase chain reaction. (E) Results of relative fluorescence intensity. **Table S1.** Clinicopathologic features for each of the subgroups. **Table S2.** Real-time PCR primers. **Table S3.** Antibodies features. **Table S4.** Sequences of siRNAs.

Figure S1

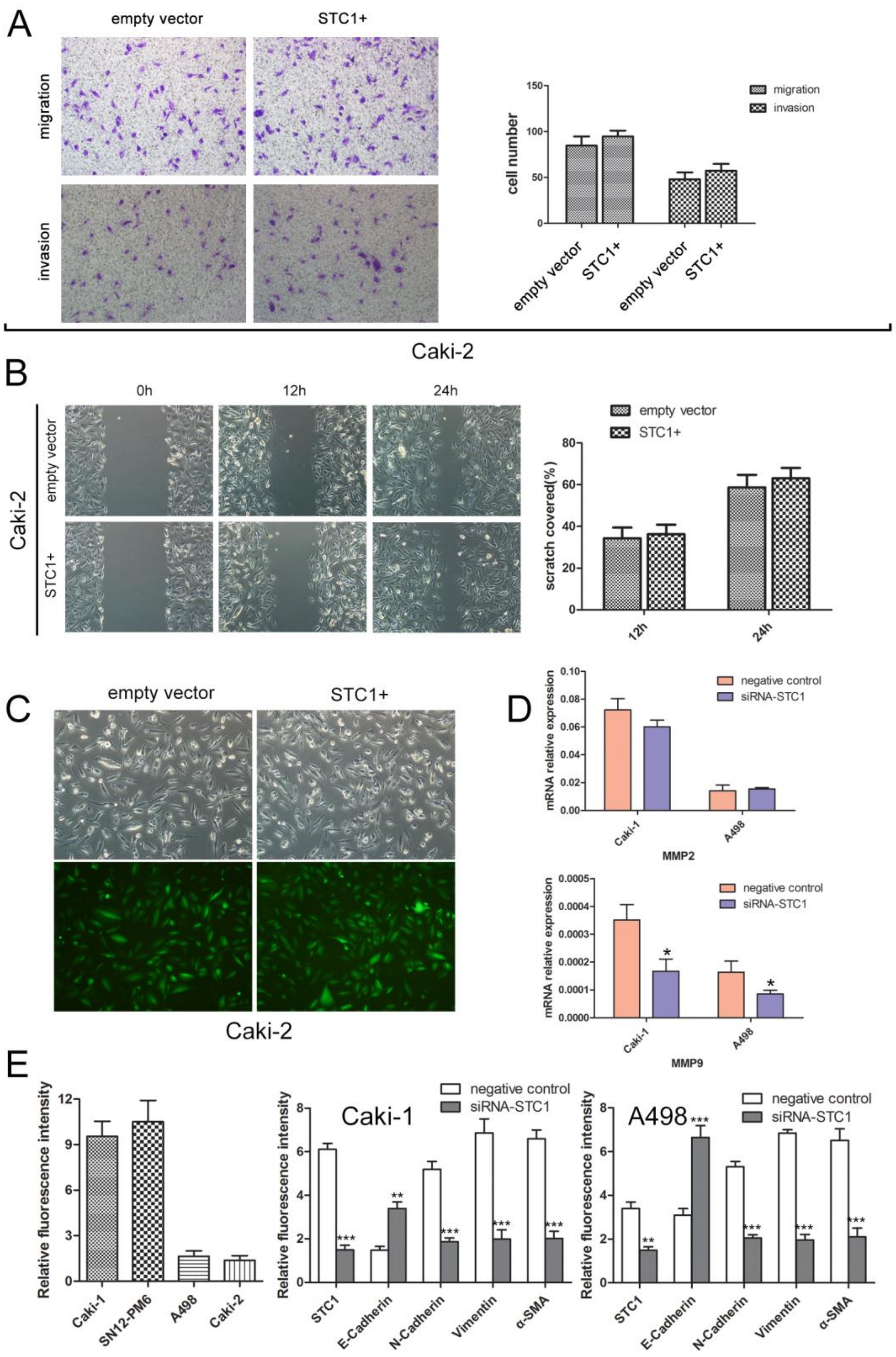
A
empty vector
STC1+
migration
invasion
cell number
migration
invasion
Caki-2
B
0h
12h
24h
empty vector
STC1+
Caki-2
scratch covered(%)
12h
24h
C
empty vector
STC1+
Caki-2
D
negative control
siRNA-STC1
mRNA relative expression
Caki-1
A498
MMP2
MMP9
E
Relative fluorescence intensity
Caki-1
SN12-PM6
A498
Caki-2
negative control
siRNA-STC1
STC1
E-Cadherin
N-Cadherin
Vimentin
α-SMA

Table S1 Clinicopathologic features for each of the subgroups

Clinicopathologic features	Localized	Metastatic	Non-tumor
	**n=122**	**n=24**	**n=48**
Age, y			
<60	86	16	32
≥60	36	8	16
Gender			
Male	79	21	42
Female	43	3	6
Tumor size, cm			
≤7	108	13	42
>7	14	11	6
Fuhrman tumor grade			
Ⅰ-Ⅱ	104	12	45
Ⅲ-Ⅳ	18	12	3
T staging			
T1+T2	107	16	41
T3	15	8	7
Overall TNM staging			
Ⅰ-Ⅱ	105	0	41
Ⅲ-Ⅳ	17	24	7
Necrosis			
Yes	61	13	25
No	61	11	23
Microvascular invasion			
Yes	14	8	7
No	108	16	41

Table S2 Real-time PCR primers

Gene	Primer Sequence	Amplicon
STC1	Forward primer: AGCAGAATGACTCTGTGAGCCC(22bp)	93bp
	Reverse primer: CGACCTGTAGAGCACTGTTGAGG(23bp)	
MMP2	Forward primer: GCGGCGGTCACAGCTACTT(19bp)	71bp
	Reverse primer: CACGCTCTTCAGACTTTGGTTCT(23bp)	
MMP9	Forward primer: CCTGGAGACCTGAGAACCAATC(22bp)	80bp
	Reverse primer: CCACCCGAGTGTAACCATAGC(21bp)	
HIF-1α	Forward primer: CCGAGGAAGAACTATGAA(18bp)	92bp
	Reverse primer: GTTGGTTACTGTTGGTATC(19bp)	
PPIA	Forward primer: ATGGTCAACCCCACCGTGT(19bp)	101bp
	Reverse primer: TCTGCTGTCTTTGGGACCTTGTC(23bp)	

Table S3 Antibodies features

Antigen	Species	Application and dilutions	Source
STC1	Goat	WB (1:1000)	R&D Systems (cat AF2958)
STC1	Goat	IHC (1:100)	Santa Cruz (cat sc-14346)
STC1	Rabbit	IF (1:50)	Santa Cruz (cat sc-30183)
E-Cadherin	Rabbit	WB (1:1000); IF (1:200)	Cell Signaling (cat 3195)
N-Cadherin	Rabbit	WB (1:1000); IF (1:100)	Abcam (cat ab76057)
Vimentin	Rabbit	WB (1:1000); IF (1:100)	Cell Signaling (cat 5741)
α-SMA	Mouse	WB (1:300); IF (1:100)	Abcam (cat ab7817)
ZEB1	Rabbit	WB (1:1000)	Cell Signaling (cat 3396)
Cyclin D1	Rabbit	WB (1:5000)	Abcam (cat ab740754)
Cdk4	Rabbit	WB (1:5000)	Abcam (cat ab108357)
Cdk6	Rabbit	WB (1:10000)	Abcam (cat ab124821)
p21	Mouse	WB (1:2000)	Cell Signaling (cat 2946)
β-actin	Mouse	WB (1:3000)	ZSGB-BIO

Table S4 List of siRNAs used in this study

Gene Name	Sense (5'-3')	Antisense (5'-3')
STC1	GGAUGUAUGACAUCUGUAATT	UUACAGAUGUCAUACAUCCTT
STC1	CCACUUUCCAAAGGAUGAUTT	AUCAUCCUUUGGAAAGUGGTT
STC1	CCCAAUCACUUCUCCAACATT	UGUUGGAGAAGUGAUUGGGTT
negative control	UUCUCCGAACGUGUCACGUTT	ACGUGACACGUUCGGAGAATT
